# Clinicopathologic characteristics, laboratory parameters, treatment protocols, and outcomes of pancreatic cancer: a retrospective cohort study of 1433 patients in China

**DOI:** 10.7717/peerj.4893

**Published:** 2018-05-28

**Authors:** Shuisheng Zhang, Xiaozhun Huang, Yuan Tian, Saderbieke Aimaiti, Jianwei Zhang, Jiuda Zhao, Yingtai Chen, Chengfeng Wang

**Affiliations:** 1 Department of Pancreatic and Gastric Surgery, National Cancer Center/Cancer Hospital, Chinese Academy of Medical Sciences and Peking Union Medical College, Beijing, China; 2 Department of Abdominal Surgery, National Cancer Center/Cancer Hospital & Shenzhen Hospital, Chinese Academy of Medical Sciences and Peking Union Medical College, Shenzhen, China; 3 Department of Radiation Oncology, Shandong Provincial Qianfoshan Hospital, Jinan, Shandong, China; 4 Department of Medical Oncology, Affiliated Hospital of Qinghai University, Xining, China

**Keywords:** Pancreatic cancer, Survival, Prognosis, Treatment, Clinicopathologic characteristic

## Abstract

**Objectives:**

The prognosis of people with pancreatic cancer is extremely unfavorable. However, the prognostic factors remain largely undefined. We aimed to perform comprehensive analyses of clinicopathologic characteristics, laboratory parameters, and treatment protocols for exploring their role as prognostic factors of pancreatic cancer.

**Methods:**

Patients diagnosed with pancreatic cancer and hospitalized at the China National Cancer Center between April 2006 and May 2016 were enrolled in this retrospective cohort study. Clinicopathologic characteristics, laboratory parameters, and treatment protocols were compared among patients at different stages of the disease. The association between these factors and overall survival (OS) was analyzed using the Kaplan–Meier method and Cox proportional hazards model.

**Results:**

The present study included 1,433 consecutive patients with pancreatic cancer. Median OS was 10.6 months (95% confidence interval [CI] 9.8–11.3 months), with 1-, 3-, and 5-year survival rates of 43.7%, 14.8%, and 8.8%, respectively. Cox multivariate analysis findings identified the following factors as independent predictors of OS: gender (female vs male, hazard ratio 0.72, 95% CI [0.54–0.95]); elevated total bilirubin (TBil; 1.82, 1.34–2.47); elevated carbohydrate antigen 19-9 (CA19-9; 1.72, 1.17–2.54); tumor being located in pancreatic body and tail (1.52, 1.10–2.10); advanced T stage (T3-4 vs T1-2, 1.62, 1.15–2.27); lymph node metastasis (1.57, 1.20–2.07); distant metastasis (1.59, 1.12–2.27); the presence of surgical resection (0.53, 0.34–0.81); and the presence of systemic chemotherapy (0.62, 0.45–0.82).

**Conclusions:**

Being male, elevated TBil and carcinoembryonic antigen, tumor being located in pancreatic body and tail, advanced T stage, lymph node and distant metastasis, the absence of surgical resection, and the absence of systematic chemotherapy were associated with worse OS in patients with pancreatic cancer.

## Introduction

Pancreatic cancer is the fourth-leading cause of cancer mortality worldwide and is estimated to become the second-leading cause by 2030 (Lucas et al., 2016; Rahib et al., 2014). In the United States, 55,440 new cases (3.2% of all cancers) and 44,330 deaths (accounting for 7.3% of all cancer-associated deaths) are estimated in 2018, which places a considerable burden on society (Siegel, Miller & Jemal, 2018).

The prognosis of pancreatic cancer is very poor, with a five-year survival rate of approximately 8% (Siegel, Miller & Jemal, 2018). Therefore, the identification of prognosis factors that can predict survival outcomes and guide proper treatment is imperative. Numerous studies (Jooste et al., 2016; Kozak et al., 2016; Sho et al., 2015; Toriola et al., 2014; Wang et al., 2016; Zhang et al., 2012, 2017) have investigated the prognostic factors of pancreatic cancer. Tumor stage (including T stage, lymph node metastasis, and distant metastasis) has been established as a significant prognostic factor (Jooste et al., 2016; Kozak et al., 2016; Lin et al., 2017; Sho et al., 2015; Wang et al., 2016; Yamamoto et al., 2015). In addition, in an attempt to identify other prognostic factors, several studies (Lin et al., 2017; Zhang et al., 2017) focused on lifestyle factors and other perioperative prognostic characteristics such as age and tumor markers. However, the association of several markers with overall survival (OS) is controversial (Jooste et al., 2016; Kozak et al., 2016; Sho et al., 2015; Toriola et al., 2014; Wang et al., 2016; Zhang et al., 2012, 2017).

Certain studies (Lin et al., 2017; Zhang et al., 2017) presented with limitations including non-informative analyses or small sample sizes and most studies (Lewis et al., 2013; Toriola et al., 2014) were conducted in Western countries. In some previous studies (Zhang et al., 2017), the effect of smoking, alcohol, and body mass index (BMI) on pancreatic cancer survival were only explored. Hence, it is essential to conduct a comprehensive study including all probable prognostic factors of pancreatic cancer survival. The primary objective of the present study was to relatively comprehensively analyze and compare clinicopathologic characteristics, laboratory tests, and treatment protocols among a relatively large cohort of 1,433 patients with pancreatic cancer in China across different tumor stages. The secondary objective was to investigate the association between these variables and OS, and to identify the independent prognostic factors of pancreatic cancer. Thus far, this is the largest cohort of Chinese patients aiming to systematically study the prognostic factors in patients with pancreatic cancer.

## Materials and Methods

### Medical ethics

This retrospective cohort study was conducted in accordance with the ethical guidelines of the 1975 Declaration of Helsinki (sixth revision, 2008) and was approved by the Ethical Committee of China National Cancer Center (no. NCC2017SF-72).

### Inclusion and exclusion criteria

Patients who were diagnosed with pancreatic cancer and hospitalized at the China National Cancer Center/Cancer Hospital between April 1, 2006 and May 31, 2016 were identified and included. The diagnoses for all patients had been confirmed by pathological or cytological examinations. For the review, all patients must have been first treated at our center and should have complete medical records.

We excluded patients with pancreatic neuroendocrine tumor, solid pseudopapillary tumor of pancreas, all kinds of pancreatitis and other benign pancreatic diseases, and so on.

### Data collection

Data on basic patient characteristics (sex, age, region of residency, race, payment method, job, marital status, lifestyle factors, comorbidities, year of diagnosis, and initial diagnostic department); clinicopathologic features (symptoms, tumor location, tumor diameter, and tumor stage); laboratory parameters (blood cell count, blood biochemical parameters, and tumor markers); and treatment information were collected from the medical records by trained investigators.

Vital status was collected using several methods. The primary methods included a telephone interview of patients or their next of kin and via Short Message Service. For patients who could not be reached, data were obtained from population registries from local health units, municipal registration offices, and local authorities. The outpatient records system was also queried for obtaining follow-up information and treatment records. All data were anonymized and de-identified.

Tumor stages were confirmed in accordance with the American Joint Committee on Cancer (AJCC) staging manual, 8th edition (Amin et al., 2017).

### Statistical analysis

Continuous variables were expressed as means ± standard deviation or medians with range, and categorical variables were expressed as frequencies and ratios. Student’s *t* test, one-way analysis of variance, the Mann–Whitney *U* test, and Kruskal–Wallis *H* test were used for comparing continuous variables among different groups. The chi-square or Fisher’s exact tests were used for comparing categorical variables among different groups. Ranked data among different groups was compared using the Mann–Whitney *U* or Kruskal–Wallis *H* tests.

Overall survival was defined as the time from the date of definite diagnosis until the death or end of follow-up. The Kaplan–Meier method and the log-rank test were used for calculating median OS rates with 95% confidence intervals (CIs), comparing OS rates among different groups, and generating survival curves. The results of univariate and multivariate analyses of OS in patients with pancreatic cancer were expressed as hazard ratios (HRs) with 95% CIs. Because some variables were repeated, the following three different models were used when we performed the Cox multivariate analyses: (1) Model 1: adjusted for gender, age, diagnosis year, and variables with *P* < 0.05 in the univariate analysis. We excluded tumor diameter because this data partly overlapped with T stage data. We also excluded total AJCC stage because this data overlapped with T stage, N stage, and M stage data. (2) Model 2: adjusted for gender, age, diagnosis year, and variables with *P* < 0.1 in the Cox multivariate analysis of Model 1. Variables included were gender, age, diagnosis year, total bilirubin (TBil), carbohydrate antigen 19-9 (CA19-9), tumor location, N stage, M stage, surgical resection, and systemic chemotherapy. We excluded T stage because this data partly overlapped with tumor diameter. (3) Model 3: adjusted for gender, age, diagnosis year, and variables with *P* < 0.1 in the Cox multivariate analysis of Model 1. Variables included were gender, age, diagnosis year, TBil, CA19-9, tumor location, surgical resection, and systemic chemotherapy. We excluded T stage, N stage, and M stage because this data overlapped with total AJCC stage data.

Statistical analyses were performed using IBM SPSS software, version 20.0 (SPSS, Chicago, IL, USA). All tests were two-sided, with a *P* value of <0.05 considered as statistically significant. And in some conditions, we set the test level at 0.1.

## Results

### Overall cohort characteristics

This retrospective cohort consisted of 1,433 pancreatic cancer patients with median follow up period of 6.3 (0–132.0) months. Overall demographic included 125 (9.0%) stage I, 157 (11.3%) stage II, 584 (42.0%) stage III, and 524 (37.7%) stage IV pancreatic cancer, respectively.

### Basic characteristics

Table 1 shows the basic characteristics of the cohort. Median age was 60 (23–90) years, and male/female ratio was 1.61:1. The majority of the patients (61.6%) were from North China, one of the six regions in China, were of Han ethnicity (93.6%), payed the treatment costs by insurance (75.7%), and were married (97.5%). Mean BMI was 23.2 kg/m^2^ and median BMI was 22.9 kg/m^2^. Compared with the other stages, the number of patients with a family history of cancer was lower in stage I or III, and the number of patients with a family history of pancreatic cancer was higher in stage II. More than 70% of patients with stage I to III cancer were admitted to the department of abdominal surgery at their first visit, while more than half of the patients with stage IV cancer were admitted to the department of internal medicine or intervention therapy (Table 1). No significant differences were noted in sex, age, region of origin, race, payment method, job, marital status, lifestyle factors (drinking, smoking, BMI) and comorbidities (hypertension, diabetes mellitus, and biliary or gallbladder disease) among the different stages of pancreatic cancer (*P* > 0.05, Table 1).

**Table 1:** Basic characteristics of patients with pancreatic cancer.

Characteristic	All patients (*n* = 1,433)	Stage I (*n* = 125, 9.0%)	Stage II (*n* = 157, 11.3%)	Stage III (*n* = 584, 42.0%)	Stage IV (*n* = 524, 37.7%)	*P* value
**Gender**						
Male	883 (61.6%)	76 (60.8%)	90 (57.3%)	351 (60.1%)	341 (65.1%)	0.215
Female	550 (38.4%)	49 (39.2%)	67 (42.7%)	233 (39.9%)	183 (34.9%)	
**Age**, median (range), years	60 (23–90)	59 (31–82)	61 (23–78)	60 (30–90)	59 (27–87)	0.234
**Region of residency**						
North China	883 (61.6%)	75 (60.0%)	95 (60.5%)	317 (63.5%)	317 (60.5%)	0.707
Other	550 (38.4%)	50 (40.0%)	62 (39.5%)	213 (36.5%)	207 (39.5%)	
**Race**						
Han	1,342 (93.6%)	115 (92.0%)	148 (94.3%)	539 (92.3%)	498 (95.0%)	0.255
Other	91 (6.4%)	10 (8.0%)	9 (5.7%)	45 (7.7%)	26 (5.0%)	
**Payment method**						
Self-payment	223 (15.6%)	14 (11.2%)	19 (12.1%)	104 (17.8%)	82 (15.6%)	0.457
Insurance	1,085 (75.7%)	99 (79.2%)	124 (79.0%)	427 (73.1%)	398 (76.0%)	
Other or unknown payment method	125 (8.7%)	12 (9.6%)	14 (8.9%)	53 (9.1%)	44 (8.4%)	
**Job**						
Retired personnel	174 (12.1%)	25 (20.0%)	23 (14.6%)	62 (10.6%)	61 (11.6%)	0.166
Officer	236 (16.5%)	18 (14.4%)	26 (16.6%)	89 (15.2%)	94 (17.9%)	
Worker and farmer	377 (26.3%)	30 (24.0%)	35 (22.3%)	157 (26.9%)	144 (27.5%)	
Other	646 (45.1%)	52 (41.6%)	73 (46.5%)	276 (47.3%)	225 (42.9%)	
**Marital status**						
Married	1,397 (97.5%)	121 (96.8%)	153 (97.5%)	567 (97.1%)	515 (98.3%)	0.510
Other (unmarried, single, or widow)	36 (2.5%)	4 (3.2%)	4 (2.5%)	17 (2.9%)	9 (1.7%)	
**Lifestyle factor**						
**Alcohol consumption**	296 (21.2%)	31 (25.4%)	32 (20.8%)	104 (18.2%)	122 (24.2%)	0.067
**Smoking**	345 (24.7%)	33 (27.0%)	41 (26.6%)	133 (23.2%)	128 (25.4%)	0.687
**Body mass index**						
Mean ± SD, kg/m^2^	23.2 ± 3.2	23.6 ± 3.4	22.8 ± 3.0	23.2 ± 3.1	23.2 ± 3.4	0.218
Median (range), kg/m^2^	22.9 (14.4–37.0)	23.5 (14.4–33.7)	22.8 (15.2–32.7)	23.1 (15.4–32.8)	22.8 (15.4–37.0)	0.468
**Comorbidity**						
Hypertension	354 (24.8%)	34 (27.2%)	37 (23.6%)	155 (26.6%)	118 (22.6%)	0.414
Diabetes mellitus	336 (23.5%)	32 (25.6%)	42 (26.8%)	144 (24.7%)	110 (21.1%)	0.337
Biliary or gallbladder disease	81 (5.7%)	9 (7.3%)	8 (5.1%)	41 (7.0%)	19 (3.6%)	0.079
Family history of cancer	183 (12.8%)	13 (10.4%)	26 (16.6%)	52 (8.9%)	85 (16.2%)	0.001
Family history of pancreatic cancer	27 (1.9%)	3 (2.4%)	9 (5.7%)	5 (0.9%)	10 (1.9%)	0.001
**Diagnosis year**						
2006–2013	1,071 (74.7%)	84 (67.2%)	94 (59.9%)	458 (78.4%)	399 (76.1%)	<0.001
2014–2016	362 (25.3%)	41 (32.8%)	63 (40.1%)	126 (21.6%)	125 (23.9%)	
**First diagnostic department**						
Department of abdominal surgery	877 (61.2%)	88 (70.4%)	115 (73.2%)	423 (72.4%)	236 (45.0%)	<0.001
Department of internal medicine	314 (21.9%)	26 (20.8%)	31 (19.7%)	101 (17.3%)	146 (27.9%)	
Department of intervention therapy	195 (13.6%)	6 (4.8%)	7 (4.5%)	39 (6.7%)	128 (24.4%)	
Other	47 (3.3%)	5 (4.0%)	4 (2.5%)	21 (3.6%)	14 (2.7%)	

**Note:**

SD, standard deviation.

### Clinical symptoms

Abdominal or back pain were observed in most patients (74.3%) at the time of diagnosis. Other common symptoms included weight loss (45.0%), jaundice (30.3%), and alimentary symptoms (14.1%). Only 116 patients (8.2%) reported no symptoms at the time of diagnosis. There was a correlation between pain and cancer stage, with pain reported more frequently by patients with advanced stage cancer. The number of patients with jaundice was lowest in stage IV (*P* < 0.001), and the number of patients with no obvious symptoms was higher in stages I and II (*P* < 0.001, Table 2).

**Table 2:** Clinicopathologic characteristics, preoperative tests, and treatment protocols of patients with pancreatic cancer.

Characteristic	All patients (*n* = 1,433)	Stage I (*n* = 125, 9.0%)	Stage II (*n* = 157, 11.3%)	Stage III (*n* = 584, 42.0%)	Stage IV (*n* = 524, 37.7%)	*P* value
**Clinical symptom**						
Pain (abdominal or back)	1,050 (74.3%)	58 (47.2%)	103 (67.3%)	438 (75.8%)	419 (80.4%)	<0.001
Jaundice	428 (30.3%)	47 (38.2%)	46 (30.1%)	239 (41.3%)	81 (15.5%)	<0.001
Alimentary symptoms	200 (14.1%)	16 (13.0%)	23 (15.0%)	87 (15.1%)	67 (12.9%)	0.723
Weight loss	637 (45.0%)	48 (39.0%)	60 (39.2%)	261 (45.2%)	250 (48.0%)	0.126
No obvious symptom	116 (8.2%)	25 (20.3%)	23 (15.0%)	23 (4.0%)	44 (8.4%)	<0.001
**Laboratory test**						
Red cell count, median (range), × 10^12^/L	4.26 (1.83–6.31)	4.30 (2.99–5.36)	4.31 (2.59–5.74)	4.24 (1.83–5.95)	4.28 (2.41–6.31)	0.504
Hemoglobin, median (range), g/L	131 (48–181)	132 (96–161)	131 (75–179)	131 (48–172)	131 (71–181)	0.863
White cell count, median (range), × 10^9^/L	6.21 (1.10–29.5)	5.87 (2.73–13.98)	6.05 (3.12–14.46)	5.98 (2.73–14.32)	6.54 (1.10–29.50)	0.004
Neutrophilic granulocyte, median (range), × 10^9^/L	3.90 (0.80–28.40)	3.63 (1.09–9.15)	3.62 (1.55–12.37)	3.73 (0.80–12.41)	4.37 (0.82–28.40)	<0.001
Lymphocyte, median (range), × 10^9^/L	1.50 (0.10–7.12)	1.56 (0.54–7.12)	1.63 (0.67–2.92)	1.48 (0.10–4.66)	1.47 (0.20–5.92)	0.017
Blood platelet, median (range), × 10^9^/L	196 (36–679)	214 (64–373)	197 (71–429)	204 (66–679)	188 (36–577)	0.001
Alanine aminotransferase, median (range), U/L	30 (1–1,137)	34 (7–548)	23 (4–465)	36 (1–1,137)	26 (4–816)	0.021
Aspartate aminotransferase, median (range), U/L	27 (3–868)	29 (8–868)	23 (8–516)	29 (3–761)	27 (9–577)	0.191
Total bilirubin, median (range), μmol/L	15.1 (1.0–926.7)	15.0 (1.8–807.3)	12.8 (1.8–679.0)	19.5 (2.0–926.7)	13.3 (1.0–617.8)	<0.001
Indirect bilirubin, median (range), μmol/L	9.2 (0.5–373.3)	9.8 (0.6–235.5)	8.0 (0.5–305.3)	10.6 (1.1–373.3)	8.6 (0.7–262.2)	0.003
Alkaline phosphatase, median (range), U/L	99 (6–2,660)	90 (26–1,061)	82 (43–1,539)	111 (25–2,660)	99 (6–1,330)	0.007
γ-glutamyl transferase, median (range), U/L	63 (3–3,469)	53 (7–2,398)	43 (7–3,297)	79 (6–3,469)	65 (3–2,199)	0.058
Albumin, median (range), g/L	39.6 (18.2–52.9)	38.9 (27.2–49.7)	39.5 (23.2–52.9)	39.4 (18.2–51.3)	40.4 (23.7–51.9)	0.048
Prealbumin, median (range), mg/dL	19 (2–60)	21 (4–40)	20 (3–60)	19 (5–51)	18 (2–60)	<0.001
C-reactive protein, median (range), mg/L	8.7 (0–491.7)	4.0 (0–307.8)	3.8 (0–152.5)	7.8 (0–360.0)	20.3 (0–491.7)	<0.001
Serum creatinine, median (range), μmol/L	62 (24–488)	65 (25–128)	64 (38–126)	60 (24–149)	63 (27–488)	0.002
Carcinoembryonic antigen, median, ng/ml	4.14	3.02	3.23	3.65	6.54	<0.001
Carbohydrate antigen 19-9, median, U/ml	270.1	116.5	106.7	221.8	810.1	<0.001
Carbohydrate antigen 242, median, U/ml	49.3	19.2	28.5	40.9	202.1	<0.001
**Tumor features**						
**Location**						
Head	849 (61.7%)	85 (69.7%)	87 (55.4%)	444 (79.9%)	208 (41.6%)	<0.001
Body and tail	528 (38.3%)	37 (30.3%)	70 (44.6%)	112 (20.1%)	292 (58.4%)	
**Diameter**, median (range), cm	4.2 (0.5–15.0)	2.9 (1.0–4.0)	4.5 (1.0–14.0)	4.2 (1.5–15.0)	4.6 (0.5–15.0)	<0.001
**T-stage**						
T1	41 (3.1%)	24 (19.2%)	10 (6.5%)	1 (0.2%)	6 (1.3%)	NA
T2	218 (16.6%)	101 (80.8%)	46 (29.7%)	11 (1.9%)	59 (13.1%)	
T3	177 (13.5%)	NA	99 (7.6%)	9 (1.5%)	68 (15.1%)	
T4	877 (66.8%)	NA	NA	561 (96.4%)	316 (70.4%)	
**N-stage**						
N0	659 (50.5%)	124 (100.0%)	71 (45.2%)	314 (55.4%)	143 (31.9%)	NA
N1-2	647 (95.5%)	NA	86 (54.8%)	253 (44.6%)	305 (68.1%)	
**M-stage**						
M0	900 (63.2%)	125 (100%)	157 (100%)	584 (100%)	NA	NA
M1	524 (36.8%)	NA	NA	NA	524 (100%)	NA
Liver metastasis	392 (27.5%)	NA	NA	NA	392 (74.8%)	NA
Abdominopelvic cavity metastasis	120 (8.4%)	NA	NA	NA	120 (22.9%)	NA
Other	108 (7.6%)	NA	NA	NA	108 (20.6%)	NA
**Treatment**						
**Antitumor treatment method**						
None*	442 (30.8%)	28 (22.4%)	37 (23.6%)	206 (35.3%)	153 (29.2%)	<0.001
Resection	142 (9.9%)	47 (37.6%)	63 (40.1%)	20 (3.4%)	11 (2.1%)	
Radiotherapy/chemotherapy	719 (50.2%)	9 (7.2%)	13 (8.3%)	345 (59.1%)	342 (65.3%)	
Resection + radiotherapy/chemotherapy	130 (9.1%)	41 (32.8%)	44 (28.0%)	13 (2.2%)	18 (3.4%)	
**Surgery**	784 (54.7%)	91 (72.8%)	115 (73.2%)	412 (70.5%)	146 (27.9%)	<0.001
Pancreaticoduodenectomy	120 (15.3%)	51 (56.0%)	39 (33.9%)	20 (4.9%)	5 (3.4%)	<0.001
Distal pancreatectomy	146 (18.6%)	33 (36.3%)	66 (57.4%)	13 (3.2%)	24 (16.4%)	
Intraoperative radiotherapy	277 (35.3%)	1 (1.1%)	0 (0.0%)	240 (58.3%)	35 (24.0%)	
Exploratory laparotomy or palliative bypass surgery	233 (29.7%)	2 (2.2%)	8 (7.0%)	138 (33.5%)	81 (55.8%)	
Other surgical methods	8 (1.0%)	4 (4.4%)	2 (1.7%)	1 (0.2%)	1 (0.7%)	
**Surgical resection**	272 (19.0%)	88 (70.4%)	107 (68.2%)	33 (5.7%)	29 (5.5%)	<0.001
**Nonsurgical antitumor treatment**	646 (45.1%)	49 (39.2%)	52 (33.1%)	190 (13.7%)	331 (63.2%)	<0.001
Systemic chemotherapy	351 (24.5%)	40 (32.0%)	39 (24.8%)	84 (14.4%)	172 (32.8%)	<0.001
Interventional therapy	250 (17.4%)	10 (8.0%)	12 (7.6%)	53 (9.1%)	166 (31.7%)	<0.001
Concurrent chemoradiotherapy	98 (6.8%)	7 (5.6%)	5 (3.2%)	75 (12.8%)	10 (1.9%)	<0.001
Extracorporeal radiotherapy	55 (3.8%)	4 (3.2%)	8 (5.1%)	27 (4.6%)	10 (1.9%)	0.064
**Biliary drainage**	562 (39.2%)	21 (16.8%)	24 (15.3%)	398 (68.2%)	108 (20.6%)	<0.001
Surgical drainage	434 (30.3%)	3 (2.4%)	7 (4.5%)	346 (59.2%)	74 (14.2%)	<0.001
Other methods	146 (10.2%)	18 (14.4%)	17 (10.8%)	68 (11.6%)	35 (6.7%)	0.011

**Notes:**

NA, not available.

* Hospitalized at the China National Cancer Center but refused any antitumor treatments or only received supportive treatment including biliary drainage.

### Laboratory parameters

The number of patients with elevated white cell and neutrophilic granulocyte counts were lowest in stage IV (*P* < 0.05), whereas the number of patients with elevated lymphocyte counts were highest in stages I and II (*P* < 0.05). Patients with metastatic pancreatic cancer had lower blood platelet counts. Alanine aminotransferase, TBil, indirect bilirubin (IBil), and alkaline phosphatase (ALP) levels significantly differed across the four stages with the highest being among stage III patients (*P* < 0.05). The level of prealbumin was the lowest in stage IV, while C-reactive protein was the highest in stage IV (*P* < 0.001). The levels of tumor markers (carcinoembryonic antigen (CEA), CA19-9, and carbohydrate antigen 242 (CA242)) were significantly higher in patients with stage IV cancer than in those with other stages of cancer (*P* < 0.05). There were no differences noted in red blood cell count and hemoglobin among the stages (*P* > 0.05, Table 2).

### Tumor features

Most tumors were located in the pancreatic head (61.7%). Median tumor diameter was 4.2 (0.5–15.0) cm. Majority of the tumors were T4 stage (66.8%). Lymph node and distant metastases were noted in 49.5% and 36.8% of patients, respectively, and liver was the most common distant metastatic organ (Table 2).

### Treatment protocols

Among the 1,433 patients, 182 (12.7%) who were hospitalized at our center refused any treatment or received supportive treatments alone (not including biliary drainage), and 784 patients (54.7%) had surgeries. Among these 784 patients, 272 (34.7%) underwent surgical resection, 277 (35.3%) received intraoperative radiotherapy, 233 (29.7%) underwent exploratory laparotomy or palliative bypass surgery, and the remaining two patients (0.3%) underwent intraoperative freezing and microwave treatments, respectively. A total of 646 patients (45.1%) received nonsurgical anticancer treatment. The most frequently performed non-surgical treatments included systemic chemotherapy (*n* = 351, 24.5%); interventional therapy (*n* = 250, 30.3%); concurrent chemoradiotherapy (*n* = 98, 6.8%); and extracorporeal radiotherapy (*n* = 55, 3.8%). Among all patients with pancreatic cancer, 39.2% underwent biliary drainage; of these, 434 underwent bypass surgery and 146 underwent non-surgical drainage (Table 2).

### Overall survival

**Figure 1:**
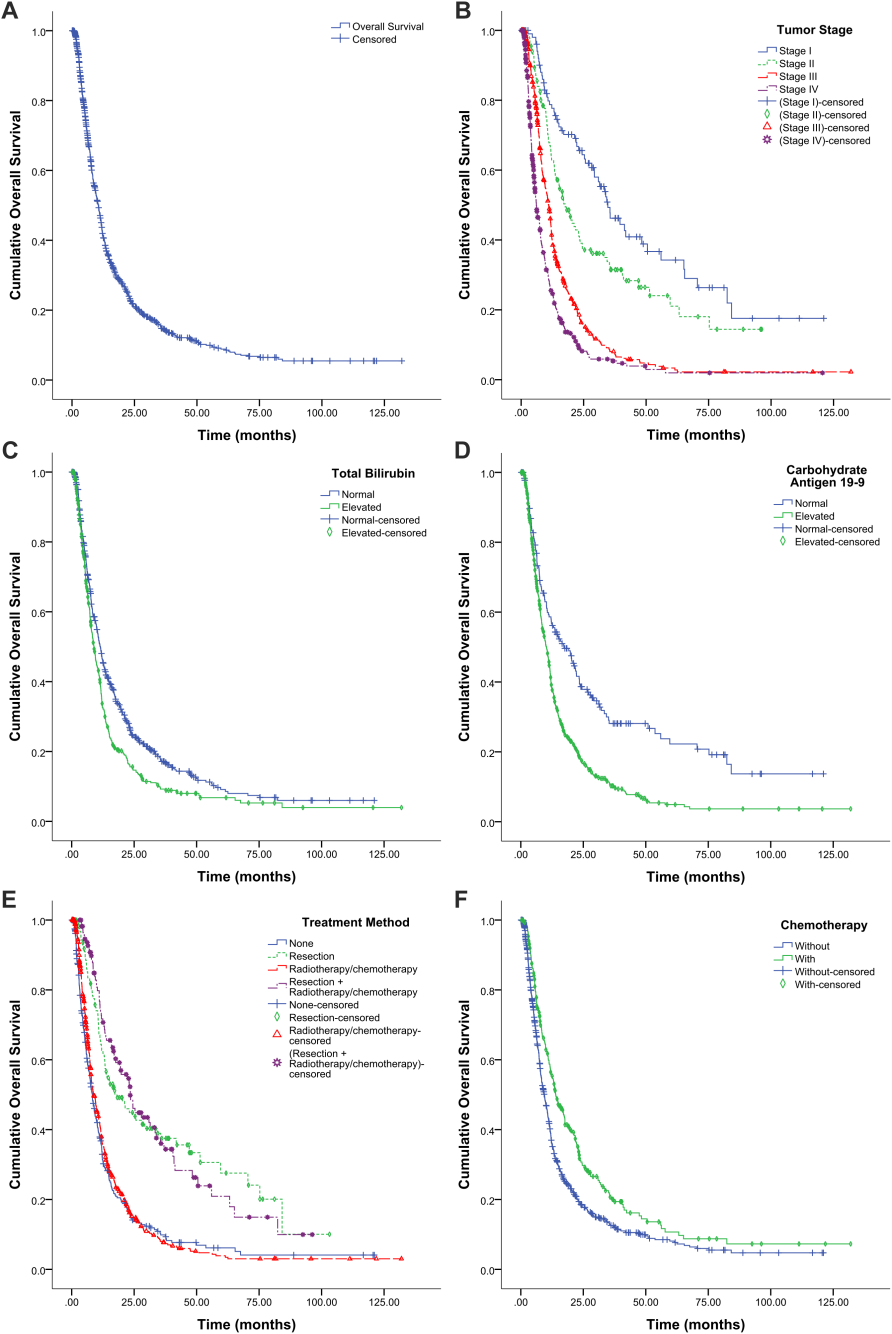
Kaplan–Meier survival graphs for overall survival (OS) in patients with pancreatic cancer. (A) OS for all patients (*n* = 1,433). (B) OS by tumor stage (log-rank test, *P* < 0.001). (C) OS by total bilirubin (log-rank test, *P* < 0.001). (D) OS by carbohydrate antigen 19-9 (log-rank test, *P* < 0.001). (E) OS by treatment protocol (log-rank test, *P* < 0.001). None, hospitalized at the study center but refused any antitumor treatments or only received supportive treatment (including biliary drainage). (F) OS by treatment with or without chemotherapy (log-rank test, *P* < 0.001).

By the end of the follow-up period, there were 874 deaths, and 342 patients remained alive or died because of non-tumor reasons. The remaining 217 patients were lost to follow-up. Overall, the 1-, 3-, and 5-year survival rates were 43.7%, 14.8%, and 8.8% respectively. Median OS was 10.6 months (95% CI, 9.8–11.3 months, Fig. 1A), and median OS rates of stages I, II, III, and IV were 34.7, 17.6, 11.0, and 6.1 months, respectively (Fig. 1B).

### Prognostic factors

We examined the association between tumor stage (Fig. 1B), laboratory parameters (Figs. 1C and 1D), and treatment protocols (Figs. 1E and 1F) and OS using the Kaplan–Meier method and the log-rank test.

Univariate analysis findings revealed that the following factors negatively affecting OS: older age (≥65 years), elevated neutrophilic granulocyte count, elevated TBil, decreased prealbumin, elevated CRP, elevated tumor biomarker levels (CEA and CA19-9), tumor being located in pancreatic body and tail, larger tumor diameter (>4 cm), higher tumor stage (total AJCC stage, T stage, N stage, and M stage), the absence of surgical resection, the absence of systemic chemotherapy, the absence of concurrent chemoradiotherapy, the presence of interventional therapy, and the presence of biliary drainage (Table 3). Using Model 1, Cox multivariate analysis found that the following factors were independent factors for OS: gender (female vs male, HR 0.72, 95% CI [0.54–0.95]), elevated TBil (HR 1.82, 95% CI [1.34–2.47]), elevated CA19-9 (HR 1.72, 95% CI [1.17–2.54]), tumor being located in pancreatic body and tail (HR 1.52, 95% CI [1.10–2.10]), advanced T stage (T3-4 vs T1-2, HR 1.62, 95% CI [1.15–2.27]), lymph node metastasis (HR 1.57, 95% CI [1.20–2.07]), distant metastasis (HR 1.59, 95% CI [1.12–2.27]), the presence of surgical resection (HR 0.53, 95% CI [0.34–0.81]), and the presence of systemic chemotherapy (HR 0.62, 95% CI [0.45–0.82]). When we set the significant level at 0.1, age (≥65 vs <65 years, HR 1.28, 95% CI [0.97–1.69]) and diagnostic time (2014–2016 vs 2006–2013, HR 0.73, 95% CI [0.53–1.001]) were also independent prognostic factors (Table 4). When adjusted using Models 2 and 3, and the significant level was set at 0.1, tumor diameter (>4 vs ≤4 cm, HR 1.17, 95% CI [0.99–1.39]) and AJCC stage (III–IV vs I–II, HR 2.10, 95% CI [1.57–2.80]) were both independent prognostic factors (Table 4).

**Table 3:** Univariate analyses of overall survival in patients with pancreatic cancer.

Characteristic	Univariate Analyses	
	**HR (95% CI)**	***P* value**
**Gender** (female vs male)	0.89 (0.78–1.02)	0.103
**Age** (≥65 vs <65 years)	1.28 (1.12–1.47)	<0.001
**Region of residency** (other regions vs North China)	1.01 (0.88–1.16)	0.893
**Race** (other races vs Han)	1.00 (0.77–1.31)	0.996
**Payment method** (insurance vs self-payment)	0.90 (0.74–1.10)	0.315
**Job** (other jobs vs officer)	1.11 (0.92–1.35)	0.301
**Marital status** (other status vs married)	1.28 (0.88–1.87)	0.199
**Lifestyle factor**		
** Alcohol consumption** (yes vs no)	1.07 (0.91–1.25)	0.429
** Smoking** (yes vs no)	1.13 (0.98–1.32)	0.099
** Body mass index** (≥24 vs <24 kg/m^2^)	0.97 (0.84–1.11)	0.637
**Comorbidity**		
** **Hypertension (yes vs no)	1.15 (0.99–1.33)	0.076
** **Diabetes mellitus (yes vs no)	1.06 (0.91–1.24)	0.476
** **Biliary or gallbladder disease (yes vs no)	0.83 (0.62–1.11)	0.211
** **Family history of cancer (yes vs no)	0.97 (0.80–1.18)	0.766
** **Family history of pancreatic cancer (yes vs no)	0.77 (0.48–1.24)	0.285
**Diagnosis year** (2014–2016 vs 2006–2013)	0.87 (0.75–1.00)	0.057
**Laboratory test**		
** **Red cell count, <3.5 vs ≥3.5 × 10^12^/L	1.24 (0.98–1.57)	0.070
** **White cell count, >10 vs ≤10 × 10^9^/L	1.42 (1.12–1.79)	0.003
** **Neutrophilic granulocyte, >7.5 vs ≤7.5 × 10^9^/L	0.56 (0.21–1.49)	0.243
** **Lymphocyte, >4 vs ≤4 × 10^9^/L	1.29 (1.00–1.66)	0.052
** **Blood platelet, >300 vs ≤300 ×10^9^/L	1.10 (0.88–1.39)	0.412
** **Total bilirubin, >17.1 vs ≤17.1 μmol/L	1.34 (1.16–1.53)	<0.001
** **Albumin, <35 vs ≥35 g/L	1.06 (0.90–1.25)	0.477
** **Prealbumin, <20 vs ≥20 mg/dL	1.29 (1.12–1.48)	<0.001
** **C-reactive protein, >10 vs ≤10 mg/L	1.40 (1.12–1.75)	0.003
** **Carcinoembryonic antigen, >5.0 vs ≤5.0 ng/ml	1.59 (1.37–1.86)	<0.001
** **Carbohydrate antigen 19-9, >37.0 vs ≤37.0 U/ml	1.79 (1.47–2.19)	<0.001
**Tumor features**		
** Location** (body and tail vs head)	1.17 (1.02–1.35)	0.023
** Diameter** (>4 vs ≤4 cm)	1.41 (1.22–1.62)	<0.001
** AJCC stage** (III–IV vs I–II)	2.94 (2.45–3.53)	<0.001
** **I	1	
** **II	1.56 (1.12–2.17)	0.009
** **III	3.11 (2.34–4.12)	<0.001
** **IV	5.01 (3.77–6.66)	<0.001
** T-stage** (T3–4 vs T1–2)	2.29 (1.90–2.76)	<0.001
** **T1	1	
** **T2	1.38 (0.86–2.21)	0.187
** **T3	1.86 (1.15–3.01)	0.012
** **T4	3.39 (2.16–5.31)	<0.001
** N-stage** (N1-2 vs N0)	1.39 (1.18–1.64)	<0.001
** M-stage** (M1 vs M0)	2.23 (1.94–2.56)	<0.001
**Treatment**		
** Surgical resection** (yes vs no)	0.41 (0.34–0.49)	<0.001
** Nonsurgical antitumor treatment** (yes vs no)		
** **Systemic chemotherapy (yes vs no)	0.68 (0.58–0.80)	<0.001
** **Interventional therapy (yes vs no)	1.39 (1.17–1.66)	<0.001
** **Concurrent chemoradiotherapy (yes vs no)	0.55 (0.42–0.72)	<0.001
** **Extracorporeal radiotherapy (yes vs no)	0.75 (0.54–1.04)	0.084
** Biliary drainage** (yes vs no)	1.19 (1.04–1.37)	0.010

**Note:**

AJCC, American Joint Committee on Cancer; CI, confidence interval; HR, hazard ratio.

**Table 4:** Multivariate analyses of overall survival in patients with pancreatic cancer.

Characteristic	Multivariate Analyses	
	HR (95% CI)	*P* value*
**Gender** (female vs male)	0.72 (0.54–0.95)	0.020
**Age** (≥65 vs <65 years)	1.28 (0.97–1.69)	0.084
**Diagnosis year** (2014–2016 vs 2006–2013)	0.73 (0.53–1.001)	0.050
**Laboratory test**		
Neutrophilic granulocyte, >7.5 vs ≤7.5 × 10^9^/L	NS	NS
Total bilirubin, >17.1 vs ≤17.1 μmol/L	1.82 (1.34–2.47)	<0.001
Prealbumin, <20 vs ≥20 mg/dL	NS	NS
C-reactive protein, >10 vs ≤10 mg/L	NS	NS
Carcinoembryonic antigen, >5.0 vs ≤5.0 ng/ml	NS	NS
Carbohydrate antigen 19-9, >37.0 vs ≤37.0 U/ml	1.72 (1.17–2.54)	0.006
**Tumor features**		
**Location** (body and tail vs head)	1.52 (1.10–2.10)	0.011
**Diameter** (>4 vs ≤4 cm)	1.17 (0.99–1.39)	0.073^#^
**AJCC stage** (III-IV vs I-II)	2.10 (1.57–2.80)	<0.001^$^
**T-stage** (T3-4 vs T1-2)	1.62 (1.15–2.27)	0.005
**N-stage** (N1-2 vs N0)	1.57 (1.20–2.07)	0.001
**M-stage** (M1 vs M0)	1.59 (1.12–2.27)	0.010
**Treatment**		
**Surgical resection** (yes vs no)	0.53 (0.34–0.81)	0.001
**Nonsurgical antitumor treatment** (yes vs no)		
Systemic chemotherapy (yes vs no)	0.62 (0.45–0.82)	0.001
Interventional therapy (yes vs no)	NS	NS
Concurrent chemoradiotherapy (yes vs no)	NS	NS
**Biliary drainage** (yes vs no)	NS	NS

**Notes:**

AJCC, American Joint Committee on Cancer; CI, confidence interval; HR, hazard ratio; NS: not significant.

* Model 1: adjusted by gender, age, diagnosis year, and variables with a *P* < 0.05 in the univariate analysis (not including tumor diameter and total AJCC stage).

# Model 2: adjusted by gender, age, diagnosis year, total bilirubin, carbohydrate antigen 19-9, tumor location, N stage, M stage, tumor resection, and systemic chemotherapy.

$ Model 3: adjusted by gender, age, diagnosis year, total bilirubin, carbohydrate antigen 19-9, tumor location, tumor resection, and systemic chemotherapy.

### Comparison between long- and short-term survivors

We also compared probable prognostic factors between long-term (≥3 years) and short-term (<3 years) survivors with pancreatic cancer. Long-term survivors were more likely to be younger and not have hypertension or diabetes mellitus as comorbidities; they also tended to not show specific symptoms of pain and weight loss and had lower levels of liver function tests (TBil, IBil, ALP, and γ-GT) and of tumor markers (CEA, CA19-9, and CA242). These long-term survivors also tended to have lower tumor stage (total AJCC, T, N, and M stages). They were also more likely to receive more antitumor treatments including surgical resection, systemic chemotherapy, and concurrent chemoradiotherapy but were less likely to undergo biliary drainage (Table 5).

**Table 5:** Comparison of prognostic factors between long-term (≥3 years) and short-term (<3 years) survivors with pancreatic cancer.

Characteristic	Short-term (<3 years) survivors (*n* = 836)	Long-term (≥3 years) survivors (*n* = 94)	*P* value
**Gender**			
Male	518 (62.0%)	54 (57.4%)	0.394
Female	318 (38.0%)	40 (42.6%)	
**Age**, median (range), years	61 (29–90)	57 (31–81)	0.013
**Region of residency**			
North China	526 (62.9%)	63 (67.0%)	0.434
Other	310 (37.1%)	31 (33.0%)	
**Race**			
Han	780 (93.3%)	89 (94.7%)	0.609
Other	56 (6.7%)	5 (5.3%)	
**Payment method**			
Self-payment	110 (13.2%)	12 (12.8%)	0.668
Insurance	640 (76.6%)	75 (79.8%)	
Other or unknown payment method	86 (10.3%)	7 (7.5%)	
**Job**			
Officer	107 (12.8%)	18 (19.1%)	0.087
Other	729 (87.2%)	76 (80.9%)	
**Marital status**			
Married	810 (96.9%)	92 (97.9%)	0.834
Other (unmarried, single, or widow)	26 (3.1%)	2 (2.1%)	
**Lifestyle factor**			
**Alcohol consumption**	180 (22.0%)	22 (23.9%)	0.676
**Smoking**	227 (27.8%)	25 (27.2%)	0.907
**Body mass index**,			
Mean ± SD, kg/m^2^	23.1 ± 3.2	23.5 ± 3.6	0.212
Median (range), kg/m^2^	22.9 (15.2–34.3)	23.0 (14.4–32.8)	0.234
**Comorbidity**			
Hypertension	228 (27.3%)	16 (17.0%)	0.031
Diabetes mellitus	208 (24.9%)	14 (14.9%)	0.030
Biliary or gallbladder disease	47 (5.6%)	6 (6.4%)	0.769
Family history of cancer	114 (13.6%)	14 (14.9%)	0.737
Family history of pancreatic cancer	15 (1.8%)	3 (3.2%)	0.388
**Clinical symptom**			
Pain (abdominal or back)	638 (76.8%)	62 (66.7%)	0.031
Jaundice	246 (29.6%)	28 (30.1%)	0.920
Alimentary symptoms	125 (15.0%)	11 (11.8%)	0.407
Weight loss	403 (48.5%)	32 (34.4%)	0.010
No obvious symptom	53 (6.4%)	16 (17.2%)	<0.001
**Laboratory test**			
Red cell count, median (range), × 10^12^/L	4.26 (1.83–5.86)	4.23 (2.99–5.45)	0.943
Hemoglobin, median (range), g/L	131 (48–181)	131 (93–166)	0.925
White cell count, median (range), × 10^9^/L	6.26 (1.10–19.03)	6.50 (2.73–16.82)	0.661
Neutrophilic granulocyte, median (range), ×10^9^/L	3.97 (0.82–15.82)	4.00 (1.09–15.48)	0.985
Lymphocyte, median (range), × 10^9^/L	1.45 (0.20–5.02)	1.67 (0.37–7.12)	0.226
Blood platelet, median (range), × 10^9^/L	197 (36–577)	199 (64–331)	0.711
Alanine aminotransferase, median (range), U/L	30 (1–816)	25 (7–482)	0.699
Aspartate aminotransferase, median (range), U/L	27 (3–868)	24 (9–386)	0.184
Total bilirubin, median (range), μmol/L	15.1 (2.8–742.9)	12.5 (1.8–403.0)	0.008
Indirect bilirubin, median (range), μmol/L	9.1 (1.1–266.0)	7.5 (0.5–196.0)	0.003
Alkaline phosphatase, median (range), U/L	100 (26–1,531)	82 (30–1,063)	0.011
γ-glutamyl transferase, median (range), U/L	66 (3–3,469)	44 (7–1,802)	0.096
Albumin, median (range), g/L	39.4 (18.2–52.9)	39.0 (25.2–47.2)	0.645
Prealbumin, median (range), mg/dL	19 (2–60)	21 (2–60)	0.309
C-reactive protein, median (range), mg/L	0.65 (0–29.50)	0.38 (0.01–11.27)	0.406
Serum creatinine, median (range), μmol/L	62 (24–488)	60 (36–128)	0.429
Carcinoembryonic antigen, median, ng/ml	4.52	2.41	<0.001
Carbohydrate antigen 19-9, median, U/ml	349.4	60.4	<0.001
Carbohydrate antigen 242, median, U/ml	61.9	17.7	0.001
**Tumor features**			
**Location**			
Head	474 (59.3%)	59 (63.4%)	0.444
Body and tail	325 (40.7%)	34 (36.6%)	
**Diameter**, median (range), cm	4.2 (0.9–15.0)	4.0 (1.0–12.0)	0.051
**AJCC stage**			
I	47 (5.8%)	30 (33.7%)	<0.001
II	81 (9.9%)	26 (29.2%)	
III	354 (43.3%)	22 (24.7%)	
IV	335 (41.0%)	11 (3.2%)	
**T-stage**			
T1	14 (1.8%)	12 (14.0%)	<0.001
T2	109 (14.1%)	27 (31.4%)	
T3	93 (12.1%)	20 (23.3%)	
T4	555 (72.0%)	27 (31.4%)	
**N-stage**			
N0	363 (47.5%)	64 (71.9%)	<0.001
N1–2	401 (52.5%)	25 (28.1%)	
**M-stage**			
M0	498 (59.8%)	83 (88.3%)	<0.001
M1	335 (40.2%)	11 (11.7%)	
Liver metastasis	249 (29.9%)	8 (8.5%)	<0.001
Abdominopelvic cavity metastasis	85 (10.2%)	2 (2.1%)	0.011
Other	72 (8.6%)	4 (4.3%)	0.142
**Treatment**			
**Surgical resection**	128 (15.3%)	48 (51.1%)	<0.001
**Nonsurgical antitumor treatment**	379 (45.3%)	47 (50.0%)	0.389
Systemic chemotherapy	202 (24.2%)	33 (35.1%)	0.021
Interventional therapy	147 (17.6%)	11 (11.7%)	0.150
Concurrent chemoradiotherapy	50 (6.0%)	11 (11.7%)	0.034
Extracorporeal radiotherapy	36 (4.3%)	4 (4.3%)	1.000
**Biliary drainage**	353 (42.2%)	25 (26.6%)	0.003
Surgical drainage	271 (32.4%)	17 (18.1%)	0.004
Other methods	92 (11.0%)	9 (9.6%)	0.673

**Note:**

AJCC, American Joint Committee on Cancer; SD, standard deviation.

For treatment, more than half (51.5%) of the long-term survivors received surgical resection, and approximately 1/3 (35.1%) received systemic chemotherapy. We failed to detect a significant difference between the long- and short-term survivors regarding interventional therapy (*P* = 0.150).

## Discussion

The incidence of pancreatic cancer in China is lower than that in the United States, and its prognosis remains poor (Chen et al., 2016; Siegel, Miller & Jemal, 2018). In the present study including 1,433 patients, the median OS was 10.6 months, with 1-, 3-, and 5-year survival rates of 43.7%, 14.8%, and 8.8% respectively. For comparison, reported five-year OS rates range from 3% to 8% (Fric et al., 2017; Kenner et al., 2017; Siegel, Miller & Jemal, 2018). Furthermore, our analysis revealed that being male, elevated TBil and CEA, tumor being located in pancreatic body and tail, advanced T stage, lymph node and distant metastasis, the absence of surgical resection, and the absence of systematic chemotherapy were associated with worse OS and served as independent prognostic factors.

Our univariate analysis revealed a positive association between older age and worse prognosis. Advanced multivariate analysis findings revealed that age had a certain association with prognosis (*P* = 0.083, we could say that older patients have a worse prognosis at a test level of 0.1), and the results were in accordance with most previous studies (Lin et al., 2017; Vernerey et al., 2016). Furthermore, we found an association between gender and OS in pancreatic cancer. Compared with female patients, male patients had a worse prognosis. Concerning the association between gender and prognosis, previous studies could not reach a consensus (Jooste et al., 2016; Kozak et al., 2016; Sho et al., 2015; Toriola et al., 2014; Wang et al., 2016; Zhang et al., 2012, 2017).

There was no significant increase in pancreatic cancer mortality among smokers, those consuming alcohol, or overweight patients. Similar findings were obtained in patients with hypertension, diabetes mellitus, or a family history of cancer. Lifestyle factors and comorbidities may not be directly associated with pancreatic cancer survival and may have varied among previous studies (Jooste et al., 2016; Kozak et al., 2016; Sho et al., 2015; Toriola et al., 2014; Wang et al., 2016; Zhang et al., 2012, 2017).

The majority of the previous studies failed to find a positive association between TBil and OS in patients with pancreatic cancer (Lin et al., 2017; Zhang et al., 2012). In a retrospective study conducted in Korea, the median OS in patients with a TBil ≥7 mg/dL was 11.4 months compared with the median OS of 14.9 months among patients with a TBil <7 mg/dL (*P* = 0.002) (Yoon et al., 2011). These findings are in accordance with the results of the present study where elevated TBil was an independent, poor prognostic factor for pancreatic cancer. In the current cohort, patients with more advanced pancreatic cancer had higher TBil levels, compared with those patients with localized tumors (45.0% vs 40.8%).

Carbohydrate antigen 19-9 is a well-known and significant diagnostic and prognostic marker for pancreatic cancer. While numerous studies demonstrated that elevated CA19-9 was associated with poor survival, which HRs reaching 9.95 (Gu et al., 2015; Lin et al., 2017), numerous other studies reported a negative association between elevated CA19-9 and survival in these patients (Kanda et al., 2014; Kim et al., 2015). In the present study, we noted that the survival rate was lower in patients with pancreatic cancer with elevated CA19-9 levels.

In addition to its diagnostic value, elevated CEA has also been proposed to be associated with poor prognosis in patients with pancreatic cancer (Lin et al., 2017; O’Brien et al., 2015). In a retrospective study in China which included 96 patients with pancreatic cancer (Lin et al., 2017), HR of pancreatic cancer mortality associated with elevated CEA reached 2.59 (95% CI [1.17–5.70]). However, several other studies found no association between CEA level and pancreatic cancer mortality (Hang et al., 2017; Tas et al., 2013), casting doubt on the prognostic value of CEA. In the present study, univariate analysis findings revealed a positive association between elevated CEA and pancreatic cancer mortality. However, Cox multivariate analysis failed to show a meaningful association between normal vs elevated CEA levels and pancreatic cancer prognosis.

Numerous studies reported a positive association between tumor stage and all-cause mortality in patients with pancreatic cancer (Jooste et al., 2016; Kozak et al., 2016; Lin et al., 2017; Sho et al., 2015; Wang et al., 2016; Yamamoto et al., 2015). A study based on the Surveillance, Epidemiology, and End Results database revealed that, compared with localized pancreatic cancer, the HRs of pancreatic cancer mortality in patients with regional infiltration and distant metastasis reached 1.89 and 3.80, respectively (Wang et al., 2016). When the role of tumor stage on outcomes was analyzed by classification according to T, N, and M stages, the HRs of pancreatic cancer mortality associated with higher T, N, and M stages reached 1.93 (Sho et al., 2015), 3.25 (Kozak et al., 2016), and 6.39 (Jooste et al., 2016), respectively. The current study confirmed the presence of a negative association between tumor stage and OS. Specifically, the present analysis was conducted with T, N, and M stages as separate variables and revealed that T, N, and M stages were all independent prognostic factors for pancreatic cancer.

Several studies have reported that tumor diameter was an important prognostic factor for pancreatic cancer (Lewis et al., 2013; Vernerey et al., 2016), agreeing with the results of our univariate analysis. Furthermore, in our advanced multivariate analysis, tumor diameter was found to have a certain association with prognosis (*P* = 0.073, we could say that patients whose tumors are larger have a worse prognosis at a test level of 0.1).

Considerable research has been undertaken to identify the association between tumor location and the prognosis of pancreatic cancer but has failed to reach a consensus (Chen et al., 2017; Kanda et al., 2014; Kim et al., 2017; van Roest et al., 2016; Yu et al., 2017). In the research conducted by Kanda et al. (2014) a total 324 patients with pancreatic cancer underwent surgical resection. Univariate analysis findings revealed that the prognosis of cancer of the pancreatic body and tail (HR 0.60) was better than cancer of the pancreatic head. However, multivariate analysis revealed that tumor location was not an independent prognostic factor for pancreatic cancer. A study by van Roest et al. included 34,757 patients. By multivariate analysis, compared with cancer of the pancreatic head, cancer of the pancreatic body and tail had a worse prognosis (HR 1.1), and the tumor location was an independent prognostic factor, which correlates with our results. A possible reason for this is that the symptoms of cancer of the pancreatic head appear earlier, enabling relatively earlier diagnosis and treatment. In the present study, compared with cancer of the pancreatic head, cancer of the pancreatic body and tail was more easily found in patients with stage IV cancer and less in patients with stage I cancer.

Surgical resection was reported to be an independent prognostic factor in pancreatic cancer in numerous studies (Mizuno et al., 2013; Singal, Singal & Kuo, 2012), correlating with our present study. Our results revealed that the median OS rates for patients with and without surgical resection were 23.0 and 8.5 months, respectively.

The prognostic value of chemotherapy varies among different studies. In some studies, chemotherapy was reported to have a beneficial effect (Amin et al., 2016; Huang et al., 2017) on pancreatic cancer survival. In one study, the HR of mortality reached 0.368 in patients treated with chemotherapy compared with those who did not receive chemotherapy (Asari et al., 2016). Conversely, some studies failed to find an association between chemotherapy and OS in pancreatic cancer (Chen et al., 2015; Kanda et al., 2014), whereas others reported a negative impact of chemotherapy on the survival of pancreatic cancer (Bergquist et al., 2016; Lee et al., 2013). In the present study, chemotherapy was a good prognostic factor for OS in pancreatic cancer.

In a retrospective study, the HR of pancreatic cancer mortality associated with interventional therapy was 0.43 (95% CI [0.29–0.43]) among 302 cases (Zhang et al., 2012); whereas, in the present study, interventional therapy failed to be an independent prognostic factor for pancreatic cancer. One possible explanation for this discrepancy may be the larger number of patients with metastatic disease (68.9%) and stage III–IV cancer (90.9%) at the time of diagnosis among who received interventional therapy than those who did not undergo interventional therapy (31.2% and 77.4%, respectively, Table 1); interventional therapy was administered to patients with later-stage disease in the current cohort. This finding highlights the requirement for special care in assessing outcomes in patients receiving interventional therapy.

Furthermore, multivariate analysis failed to identify concurrent chemoradiotherapy and extracorporeal radiotherapy as independent prognostic factors for pancreatic cancer. A possible reason is that the number of patients who received chemoradiotherapy and extracorporeal radiotherapy was relatively small (6.8% and 3.8%, respectively).

In the present study, the comparison of potential prognostic factors between long- and short-term survivors revealed that higher serum TBil, higher serum CA19-9, advanced T stage, presence of lymph node metastasis, the presence of distant metastasis, the absence of surgical resection, and the absence of systemic chemotherapy were associated with worse outcomes, which again highlights the prognostic value of these independent factors.

### Limitations and strengths

This study has several pertinent limitations. First, there may be confounding factors that could influence the results because of the retrospective design of the study. Second, this was a single-center study. Third, detailed data on chemotherapy specifics such as single vs multi drugs were not collected, and prognostic analyses of chemotherapy characteristics could not be performed. Fourth, prognostic analyses of subgroups were not performed. Some factors, such as interventional therapy, may be associated with better OS in specific patient subgroups, which we plan to address with future study series. Nonetheless, several notable strengths of this study included the analysis of a relatively large cohort (large diameter) and comprehensive analysis of a wide variety of factors that may have association with OS in pancreatic cancer, which should provide an important reference point for clinicians.

## Conclusions

Being male, elevated TBil and CEA, tumor location in the pancreatic body and tail, advanced T stage, lymph node and distant metastasis, the absence of surgical resection, and the absence of systematic chemotherapy were independent prognostic factors for pancreatic cancer, contributing to worse OS.

## Supplemental Information

10.7717/peerj.4893/supp-1 Supplemental Information 1 Dataset 1. Raw data exported from the medical records of each patient in the whole cohort.

## Additional Information and Declarations

### Data Availability

The following information was supplied regarding data availability: The raw data are provided as a Supplemental File.
